# 6-(4-Methyl­phen­yl)-1,3,5-triazine-2,4-di­amine–4-methyl­benzoic acid (1/1)

**DOI:** 10.1107/S1600536813013895

**Published:** 2013-05-25

**Authors:** Kaliyaperumal Thanigaimani, Suhana Arshad, Ibrahim Abdul Razak, Duraisamy Makeshvaran, Kasthuri Balasubramani

**Affiliations:** a School of Physics, Universiti Sains Malaysia, 11800 USM, Penang, Malaysia; b Department of Chemistry, Government Arts College (Autonomous), Thanthonimalai, Karur 639 005, Tamil Nadu, India

## Abstract

The 4-methyl­benzoic acid mol­ecule of the title adduct, C_10_H_11_N_5_·C_8_H_8_O_2_, is approximately planar with a dihedral angle of 6.3 (2)° between the carb­oxy­lic acid group and the benzene ring. In the triazine mol­ecule, the plane of the triazine ring makes a dihedral angle of 29.2 (2)° with that of the adjacent benzene ring. In the crystal, the acid and base mol­ecules are linked *via* N—H⋯O and O—H⋯N hydrogen bonds with an *R*_2_^2^(8) motif, and the acid–base pairs are further connected *via* N—H⋯N hydrogen bonds with *R*_2_^2^(8) motifs, forming a supra­molecular ribbon along [101]. Between the tapes, a weak C—H⋯π inter­action is observed.

## Related literature

The background to this study has been described in the preceding paper, see: Thanigaimani *et al.* (2013 ▶). For hydrogen-bond motifs, see: Bernstein *et al.* (1995 ▶). For bond-length data, see: Allen *et al.* (1987 ▶). For stability of the temperature controller used for the data collection, see: Cosier & Glazer (1986 ▶).
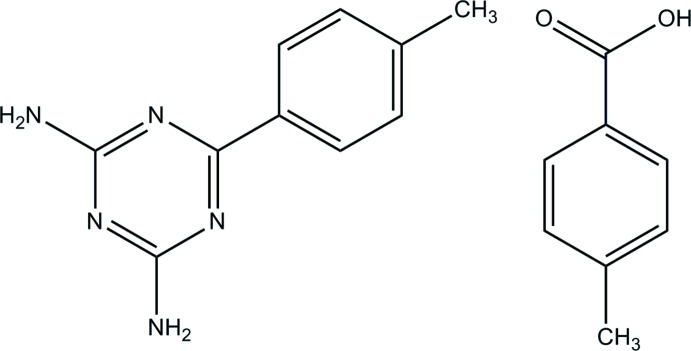


## Experimental

### 

#### Crystal data

C_10_H_11_N_5_·C_8_H_8_O_2_
*M* *_r_* = 337.38
Monoclinic, 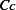

*a* = 11.1271 (3) Å
*b* = 20.9492 (6) Å
*c* = 7.4189 (2) Å
β = 101.321 (2)°
*V* = 1695.73 (8) Å^3^
*Z* = 4
Mo *K*α radiation
μ = 0.09 mm^−1^
*T* = 100 K
0.40 × 0.40 × 0.20 mm

#### Data collection

Bruker SMART APEXII CCD area-detector diffractometer
Absorption correction: multi-scan (*SADABS*; Bruker, 2009 ▶) *T*_min_ = 0.965, *T*_max_ = 0.982
8715 measured reflections
1932 independent reflections
1811 reflections with *I* > 2σ(*I*)
*R*_int_ = 0.031

#### Refinement

*R*[*F* ^2^ > 2σ(*F* ^2^)] = 0.055
*wR*(*F* ^2^) = 0.135
*S* = 1.19
1932 reflections
244 parameters
3 restraints
H atoms treated by a mixture of independent and constrained refinement
Δρ_max_ = 0.27 e Å^−3^
Δρ_min_ = −0.27 e Å^−3^

### 

Data collection: *APEX2* (Bruker, 2009 ▶); cell refinement: *SAINT* (Bruker, 2009 ▶); data reduction: *SAINT*; program(s) used to solve structure: *SHELXTL* (Sheldrick, 2008 ▶); program(s) used to refine structure: *SHELXTL*; molecular graphics: *SHELXTL*; software used to prepare material for publication: *SHELXTL* and *PLATON* (Spek, 2009 ▶).

## Supplementary Material

Crystal structure: contains datablock(s) global, I. DOI: 10.1107/S1600536813013895/is5272sup1.cif

Structure factors: contains datablock(s) I. DOI: 10.1107/S1600536813013895/is5272Isup2.hkl

Supplementary material file. DOI: 10.1107/S1600536813013895/is5272Isup3.cml

Additional supplementary materials:  crystallographic information; 3D view; checkCIF report

## Figures and Tables

**Table 1:** Hydrogen-bond geometry (Å, °) *Cg*2 is the centroid of the C4–C9 ring.

*D*—H⋯*A*	*D*—H	H⋯*A*	*D*⋯*A*	*D*—H⋯*A*
O2—H1*O*2⋯N2	0.77 (6)	1.92 (6)	2.682 (4)	171 (6)
N4—H1*N*4⋯O1	0.97 (6)	1.98 (6)	2.936 (5)	169 (6)
N4—H2*N*4⋯N3^i^	0.84 (4)	2.26 (4)	3.099 (5)	172 (4)
N5—H1*N*5⋯N1^ii^	0.90 (6)	2.11 (6)	3.003 (5)	173 (5)
C10—H10*C*⋯*Cg*2^iii^	0.98	2.83	3.723 (6)	152

Symmetry codes: (i) 
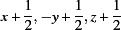
; (ii) 
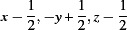
; (iii) 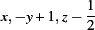.

**Fig. 1.**
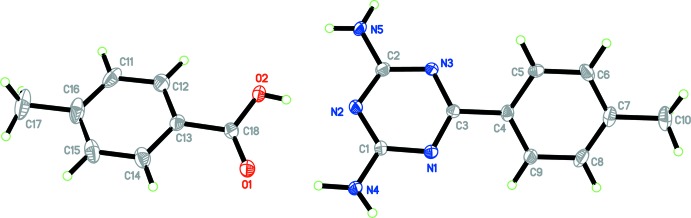
The molecular structure of the title compound with atom labels with 50% probability displacement ellipsoids.

**Fig. 2.**
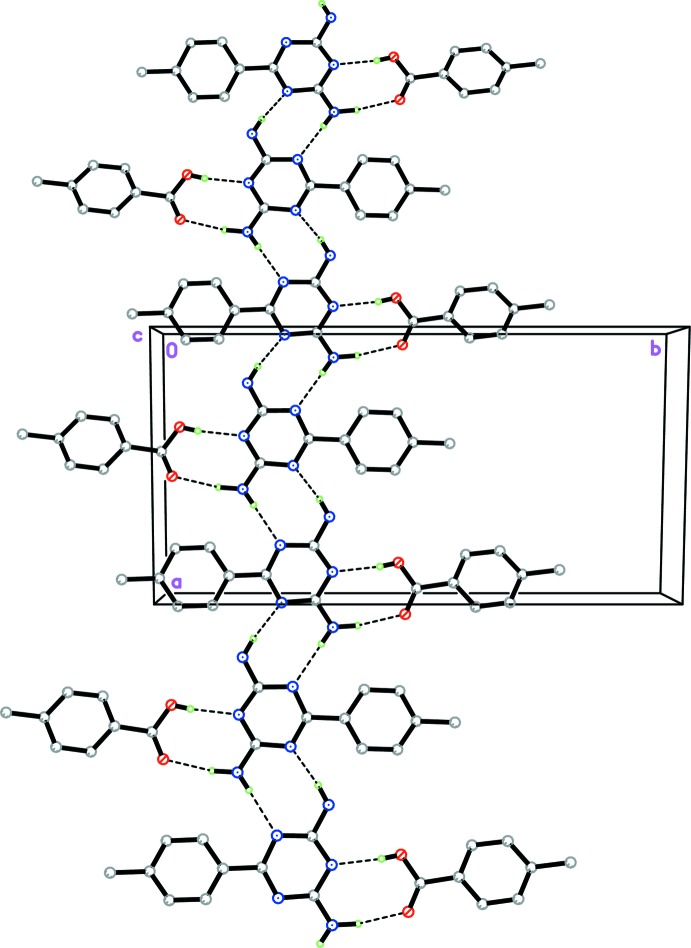
The crystal packing of the title compound. The H atoms not involved in the intermolecular interactions (dashed lines) have been omitted for clarity.

## supplementary crystallographic information

### Comment

This work follows on from our previous report on 2,4-diamino-6-(4-methylphenyl)-1,3,5-triazine–benzoic acid (1/1) (Thanigaimani *et al.*, 2013).

The asymmetric unit (Fig. 1) contains one 2,4-diamino-6-(4-methylphenyl)- 1,3,5-triazine molecule and one 4-methylbenzoic acid molecule. The dihedral angle between the triazine ring [N1/C1/N2/C2/N3/C3, maximum deviation = 0.003 (4) Å for atom N1] and the plane formed by the benzoic acid molecule (O1/O2/C11–C18) is 12.04 (19)°. The triazine ring forms a dihedral angle of 29.2 (2)° with the benzene ring (C4–C10). The bond lengths (Allen *et al.*, 1987) and angles are normal.

In the crystal (Fig. 2), the triazine molecules are base-paired with a *R*_2_^2^(8) graph-set motifs (Bernstein *et al.*, 1995) on either side *via* N4—H2N4···N3^ii^ and N5—H1N5···N1^i^ hydrogen bonds (symmetry codes in Table 1) and further interact with the carboxyl group of 4-methylbenzoic acid molecules *via* N4—H1N4···O1 and O2—H1O2···N2 hydrogen bonds, forming an *R*_2_^2^(8) motif and a supramolecular ribbon along the [101]. In addition, the crystal structure is stabilized by weak C—H···π interactions (Table 1) involving the C4–C9 (centroid C*g*2) ring.

### Experimental

Hot methanol solutions (20 ml) of 2,4-diamino-6-(4-methylphenyl)-1,3,5-triazine (50 mg Aldrich) and 4-methylbenzoic acid (34 mg Aldrich) were mixed and warmed over a heating magnetic stirrer hotplate for a few minutes. The resulting solution was allowed to cool slowly at room temperature and crystals of the title compound (I) appeared after a few days.

### Refinement

O- and N-bound H atoms were located in a difference Fourier maps. The O-bound H atom was refined freely [refined distance: O2—H1O2 = 0.77 (6) Å], while for the N-bound H atoms the positions were refined with *U*_iso_(H) = 1.5*U*_eq_(N). [refined distances: N5—H1N5 = 0.90 (6) Å, N5—H2N5 = 0.89 (6) Å, N4—H1N4 = 0.97 (6) Å, N4—H2N4 = 0.849 (11) Å]. A bond-length restraint of N—H = 0.85 (1) Å was also applied for N4—H2N4. The remaining hydrogen atoms were positioned geometrically (C—H = 0.95–0.98 Å) and were refined using a riding model, with *U*_iso_(H) = 1.2*U*_eq_(C) or 1.5*U*_eq_(methyl C). A rotating-group model was used for the methyl group. In the final refinement, 1279 Friedel pairs were merged.

### Crystal data

**Table:**

C_10_H_11_N_5_·C_8_H_8_O_2_	*F*(000) = 712
*M**_r_* = 337.38	*D*_x_ = 1.322 Mg m^−^^3^
Monoclinic, *C**c*	Mo *K*α radiation, λ = 0.71073 Å
Hall symbol: C -2yc	Cell parameters from 6815 reflections
*a* = 11.1271 (3) Å	θ = 3.2–33.7°
*b* = 20.9492 (6) Å	µ = 0.09 mm^−^^1^
*c* = 7.4189 (2) Å	*T* = 100 K
β = 101.321 (2)°	Plate, colourless
*V* = 1695.73 (8) Å^3^	0.40 × 0.40 × 0.20 mm
*Z* = 4	

### Data collection

**Table:**

Bruker SMART APEXII CCD area-detector diffractometer	1932 independent reflections
Radiation source: fine-focus sealed tube	1811 reflections with *I* > 2σ(*I*)
Graphite monochromator	*R*_int_ = 0.031
φ and ω scans	θ_max_ = 27.5°, θ_min_ = 1.9°
Absorption correction: multi-scan (*SADABS*; Bruker, 2009)	*h* = −14→14
*T*_min_ = 0.965, *T*_max_ = 0.982	*k* = −27→27
8715 measured reflections	*l* = −9→7

### Refinement

**Table:**

Refinement on *F*^2^	Primary atom site location: structure-invariant direct methods
Least-squares matrix: full	Secondary atom site location: difference Fourier map
*R*[*F*^2^ > 2σ(*F*^2^)] = 0.055	Hydrogen site location: inferred from neighbouring sites
*wR*(*F*^2^) = 0.135	H atoms treated by a mixture of independent and constrained refinement
*S* = 1.19	*w* = 1/[σ^2^(*F*_o_^2^) + (0.0311*P*)^2^ + 4.4301*P*] where *P* = (*F*_o_^2^ + 2*F*_c_^2^)/3
1932 reflections	(Δ/σ)_max_ < 0.001
244 parameters	Δρ_max_ = 0.27 e Å^−^^3^
3 restraints	Δρ_min_ = −0.27 e Å^−^^3^

### Special details

**Table:**

Experimental. The crystal was placed in the cold stream of an Oxford Cryosystems Cobra open-flow nitrogen cryostat (Cosier & Glazer, 1986) operating at 100.0 (1) K.
Geometry. All e.s.d.'s (except the e.s.d. in the dihedral angle between two l.s. planes) are estimated using the full covariance matrix. The cell e.s.d.'s are taken into account individually in the estimation of e.s.d.'s in distances, angles and torsion angles; correlations between e.s.d.'s in cell parameters are only used when they are defined by crystal symmetry. An approximate (isotropic) treatment of cell e.s.d.'s is used for estimating e.s.d.'s involving l.s. planes.
Refinement. Refinement of *F*^2^ against ALL reflections. The weighted *R*-factor *wR* and goodness of fit *S* are based on *F*^2^, conventional *R*-factors *R* are based on *F*, with *F* set to zero for negative *F*^2^. The threshold expression of *F*^2^ > σ(*F*^2^) is used only for calculating *R*-factors(gt) *etc*. and is not relevant to the choice of reflections for refinement. *R*-factors based on *F*^2^ are statistically about twice as large as those based on *F*, and *R*- factors based on ALL data will be even larger.

### Fractional atomic coordinates and isotropic or equivalent isotropic displacement parameters (Å^2^)

**Table:**

	*x*	*y*	*z*	*U*_iso_*/*U*_eq_	
N1	0.4994 (3)	0.25996 (17)	0.3556 (5)	0.0165 (8)	
N2	0.3884 (4)	0.16295 (15)	0.2802 (6)	0.0170 (7)	
N3	0.2919 (3)	0.26326 (16)	0.1933 (6)	0.0164 (7)	
N4	0.5874 (3)	0.16186 (18)	0.4348 (5)	0.0200 (8)	
H1N4	0.581 (5)	0.116 (3)	0.447 (8)	0.030*	
H2N4	0.646 (3)	0.183 (2)	0.496 (7)	0.030*	
N5	0.1876 (3)	0.16862 (19)	0.1268 (6)	0.0235 (9)	
H1N5	0.126 (5)	0.188 (3)	0.049 (9)	0.035*	
H2N5	0.188 (5)	0.126 (3)	0.120 (8)	0.035*	
C1	0.4899 (4)	0.19523 (19)	0.3563 (6)	0.0156 (8)	
C2	0.2928 (4)	0.19889 (19)	0.2015 (6)	0.0169 (8)	
C3	0.3982 (4)	0.29042 (16)	0.2738 (7)	0.0150 (7)	
C4	0.4034 (4)	0.36131 (17)	0.2713 (7)	0.0166 (8)	
C5	0.2976 (4)	0.3973 (2)	0.2713 (6)	0.0189 (9)	
H5A	0.2217	0.3762	0.2690	0.023*	
C6	0.3023 (4)	0.4632 (2)	0.2746 (7)	0.0228 (10)	
H6A	0.2296	0.4870	0.2744	0.027*	
C7	0.4126 (4)	0.49501 (18)	0.2781 (7)	0.0216 (9)	
C8	0.5170 (4)	0.4596 (2)	0.2779 (7)	0.0236 (10)	
H8A	0.5926	0.4809	0.2798	0.028*	
C9	0.5136 (4)	0.39311 (19)	0.2749 (6)	0.0187 (9)	
H9A	0.5865	0.3695	0.2753	0.022*	
C10	0.4170 (5)	0.5672 (2)	0.2789 (8)	0.0306 (11)	
H10A	0.4988	0.5814	0.3396	0.046*	
H10B	0.3558	0.5838	0.3455	0.046*	
H10C	0.3991	0.5830	0.1521	0.046*	
O1	0.5390 (3)	0.02412 (14)	0.4355 (5)	0.0245 (7)	
O2	0.3551 (3)	0.03627 (15)	0.2514 (5)	0.0248 (7)	
C11	0.3135 (5)	−0.1599 (2)	0.1798 (7)	0.0298 (12)	
H11A	0.2450	−0.1770	0.0970	0.036*	
C12	0.3269 (4)	−0.0939 (2)	0.1967 (7)	0.0253 (10)	
H12A	0.2683	−0.0662	0.1258	0.030*	
C13	0.4271 (4)	−0.0689 (2)	0.3187 (6)	0.0201 (10)	
C14	0.5126 (5)	−0.1101 (2)	0.4226 (7)	0.0268 (10)	
H14A	0.5807	−0.0934	0.5069	0.032*	
C15	0.4970 (5)	−0.1754 (2)	0.4014 (7)	0.0305 (12)	
H15A	0.5557	−0.2033	0.4711	0.037*	
C16	0.3977 (6)	−0.20104 (19)	0.2808 (10)	0.0338 (12)	
C17	0.3816 (7)	−0.2731 (2)	0.2623 (12)	0.0459 (16)	
H17A	0.3450	−0.2838	0.1348	0.069*	
H17B	0.3278	−0.2879	0.3437	0.069*	
H17C	0.4617	−0.2939	0.2967	0.069*	
C18	0.4468 (4)	0.0016 (2)	0.3420 (6)	0.0202 (9)	
H1O2	0.370 (6)	0.072 (3)	0.253 (9)	0.038 (16)*	

### Atomic displacement parameters (Å^2^)

**Table:**

	*U*^11^	*U*^22^	*U*^33^	*U*^12^	*U*^13^	*U*^23^
N1	0.0140 (17)	0.0145 (17)	0.0190 (18)	−0.0002 (14)	−0.0018 (15)	−0.0001 (15)
N2	0.0159 (16)	0.0136 (14)	0.0195 (15)	0.0007 (15)	−0.0012 (13)	−0.0001 (17)
N3	0.0125 (15)	0.0127 (17)	0.0224 (18)	0.0006 (14)	−0.0002 (14)	0.0021 (15)
N4	0.0155 (17)	0.0140 (17)	0.027 (2)	−0.0007 (14)	−0.0055 (15)	−0.0017 (15)
N5	0.0146 (18)	0.0144 (19)	0.037 (2)	−0.0030 (14)	−0.0063 (17)	0.0027 (16)
C1	0.0154 (18)	0.0128 (18)	0.0183 (19)	0.0004 (16)	0.0024 (16)	0.0000 (17)
C2	0.0151 (18)	0.017 (2)	0.018 (2)	−0.0021 (17)	0.0015 (16)	0.0000 (18)
C3	0.0138 (17)	0.0147 (16)	0.0168 (17)	0.0002 (17)	0.0041 (14)	−0.0008 (19)
C4	0.0211 (19)	0.0130 (16)	0.0141 (18)	0.0003 (18)	−0.0009 (15)	−0.0020 (19)
C5	0.0129 (19)	0.020 (2)	0.021 (2)	−0.0008 (16)	−0.0049 (16)	−0.0015 (17)
C6	0.022 (2)	0.019 (2)	0.024 (2)	0.0083 (18)	−0.0040 (18)	−0.0040 (18)
C7	0.032 (2)	0.0132 (17)	0.0169 (18)	−0.0022 (19)	−0.0026 (18)	0.000 (2)
C8	0.025 (2)	0.017 (2)	0.027 (2)	−0.0084 (17)	0.0001 (19)	0.0010 (18)
C9	0.018 (2)	0.0148 (19)	0.021 (2)	−0.0005 (16)	−0.0018 (17)	−0.0002 (17)
C10	0.047 (3)	0.0130 (18)	0.030 (2)	0.002 (2)	0.003 (2)	0.001 (2)
O1	0.0219 (16)	0.0158 (13)	0.0323 (18)	−0.0005 (13)	−0.0035 (13)	0.0004 (14)
O2	0.0205 (15)	0.0135 (14)	0.037 (2)	−0.0021 (12)	−0.0022 (14)	0.0019 (14)
C11	0.031 (3)	0.024 (2)	0.039 (3)	−0.013 (2)	0.019 (2)	−0.015 (2)
C12	0.025 (2)	0.020 (2)	0.034 (3)	0.0007 (18)	0.014 (2)	−0.007 (2)
C13	0.019 (2)	0.0170 (19)	0.028 (3)	−0.0016 (16)	0.0145 (19)	−0.0006 (17)
C14	0.032 (3)	0.019 (2)	0.033 (3)	0.0043 (19)	0.015 (2)	0.003 (2)
C15	0.041 (3)	0.020 (2)	0.036 (3)	0.008 (2)	0.020 (2)	0.009 (2)
C16	0.052 (3)	0.0146 (19)	0.045 (3)	−0.001 (2)	0.034 (2)	−0.005 (3)
C17	0.074 (4)	0.017 (2)	0.058 (4)	−0.008 (3)	0.041 (3)	−0.005 (3)
C18	0.019 (2)	0.0158 (19)	0.026 (2)	0.0017 (17)	0.0062 (18)	−0.002 (2)

### Geometric parameters (Å, º)

**Table:**

N1—C3	1.333 (5)	C9—H9A	0.9500
N1—C1	1.360 (5)	C10—H10A	0.9800
N2—C2	1.340 (5)	C10—H10B	0.9800
N2—C1	1.342 (6)	C10—H10C	0.9800
N3—C3	1.342 (5)	O1—C18	1.215 (5)
N3—C2	1.350 (5)	O2—C18	1.324 (5)
N4—C1	1.326 (5)	O2—H1O2	0.77 (6)
N4—H1N4	0.97 (6)	C11—C16	1.380 (8)
N4—H2N4	0.849 (11)	C11—C12	1.395 (6)
N5—C2	1.351 (5)	C11—H11A	0.9500
N5—H1N5	0.90 (6)	C12—C13	1.393 (6)
N5—H2N5	0.89 (6)	C12—H12A	0.9500
C3—C4	1.487 (5)	C13—C14	1.398 (6)
C4—C9	1.390 (6)	C13—C18	1.498 (6)
C4—C5	1.398 (6)	C14—C15	1.384 (6)
C5—C6	1.382 (6)	C14—H14A	0.9500
C5—H5A	0.9500	C15—C16	1.386 (8)
C6—C7	1.392 (7)	C15—H15A	0.9500
C6—H6A	0.9500	C16—C17	1.523 (6)
C7—C8	1.378 (6)	C17—H17A	0.9800
C7—C10	1.512 (5)	C17—H17B	0.9800
C8—C9	1.394 (6)	C17—H17C	0.9800
C8—H8A	0.9500		
			
C3—N1—C1	114.8 (3)	C8—C9—H9A	120.0
C2—N2—C1	115.5 (3)	C7—C10—H10A	109.5
C3—N3—C2	113.9 (3)	C7—C10—H10B	109.5
C1—N4—H1N4	120 (3)	H10A—C10—H10B	109.5
C1—N4—H2N4	116 (4)	C7—C10—H10C	109.5
H1N4—N4—H2N4	122 (5)	H10A—C10—H10C	109.5
C2—N5—H1N5	123 (4)	H10B—C10—H10C	109.5
C2—N5—H2N5	119 (4)	C18—O2—H1O2	113 (5)
H1N5—N5—H2N5	115 (5)	C16—C11—C12	121.4 (5)
N4—C1—N2	117.9 (4)	C16—C11—H11A	119.3
N4—C1—N1	118.0 (4)	C12—C11—H11A	119.3
N2—C1—N1	124.1 (4)	C13—C12—C11	119.2 (5)
N2—C2—N3	125.4 (4)	C13—C12—H12A	120.4
N2—C2—N5	117.7 (4)	C11—C12—H12A	120.4
N3—C2—N5	116.9 (4)	C12—C13—C14	119.9 (4)
N1—C3—N3	126.3 (3)	C12—C13—C18	121.7 (4)
N1—C3—C4	116.9 (4)	C14—C13—C18	118.5 (4)
N3—C3—C4	116.8 (3)	C15—C14—C13	119.4 (5)
C9—C4—C5	118.8 (3)	C15—C14—H14A	120.3
C9—C4—C3	121.0 (4)	C13—C14—H14A	120.3
C5—C4—C3	120.2 (4)	C14—C15—C16	121.5 (5)
C6—C5—C4	120.6 (4)	C14—C15—H15A	119.2
C6—C5—H5A	119.7	C16—C15—H15A	119.2
C4—C5—H5A	119.7	C11—C16—C15	118.6 (4)
C5—C6—C7	120.6 (4)	C11—C16—C17	121.0 (6)
C5—C6—H6A	119.7	C15—C16—C17	120.5 (6)
C7—C6—H6A	119.7	C16—C17—H17A	109.5
C8—C7—C6	118.9 (4)	C16—C17—H17B	109.5
C8—C7—C10	120.7 (4)	H17A—C17—H17B	109.5
C6—C7—C10	120.4 (4)	C16—C17—H17C	109.5
C7—C8—C9	121.2 (4)	H17A—C17—H17C	109.5
C7—C8—H8A	119.4	H17B—C17—H17C	109.5
C9—C8—H8A	119.4	O1—C18—O2	123.8 (4)
C4—C9—C8	120.0 (4)	O1—C18—C13	122.5 (4)
C4—C9—H9A	120.0	O2—C18—C13	113.7 (4)
			
C2—N2—C1—N4	−179.5 (4)	C5—C6—C7—C10	179.1 (5)
C2—N2—C1—N1	−0.5 (7)	C6—C7—C8—C9	−0.2 (8)
C3—N1—C1—N4	179.7 (4)	C10—C7—C8—C9	−179.2 (4)
C3—N1—C1—N2	0.7 (7)	C5—C4—C9—C8	−0.2 (7)
C1—N2—C2—N3	0.3 (7)	C3—C4—C9—C8	−178.1 (4)
C1—N2—C2—N5	−178.5 (4)	C7—C8—C9—C4	0.3 (7)
C3—N3—C2—N2	−0.3 (7)	C16—C11—C12—C13	−0.2 (7)
C3—N3—C2—N5	178.5 (4)	C11—C12—C13—C14	−0.1 (7)
C1—N1—C3—N3	−0.7 (8)	C11—C12—C13—C18	179.6 (4)
C1—N1—C3—C4	179.3 (4)	C12—C13—C14—C15	0.6 (7)
C2—N3—C3—N1	0.5 (8)	C18—C13—C14—C15	−179.2 (5)
C2—N3—C3—C4	−179.5 (4)	C13—C14—C15—C16	−0.7 (8)
N1—C3—C4—C9	28.0 (7)	C12—C11—C16—C15	0.2 (8)
N3—C3—C4—C9	−152.0 (5)	C12—C11—C16—C17	179.5 (5)
N1—C3—C4—C5	−149.9 (5)	C14—C15—C16—C11	0.3 (9)
N3—C3—C4—C5	30.1 (7)	C14—C15—C16—C17	−179.0 (6)
C9—C4—C5—C6	0.1 (7)	C12—C13—C18—O1	−173.4 (4)
C3—C4—C5—C6	178.0 (4)	C14—C13—C18—O1	6.4 (7)
C4—C5—C6—C7	−0.1 (7)	C12—C13—C18—O2	6.4 (6)
C5—C6—C7—C8	0.1 (8)	C14—C13—C18—O2	−173.8 (4)

### Hydrogen-bond geometry (Å, º)

*Cg*2 is the centroid of the C4–C9 ring.

**Table:**

*D*—H···*A*	*D*—H	H···*A*	*D*···*A*	*D*—H···*A*
O2—H1*O*2···N2	0.77 (6)	1.92 (6)	2.682 (4)	171 (6)
N4—H1*N*4···O1	0.97 (6)	1.98 (6)	2.936 (5)	169 (6)
N4—H2*N*4···N3^i^	0.84 (4)	2.26 (4)	3.099 (5)	172 (4)
N5—H1*N*5···N1^ii^	0.90 (6)	2.11 (6)	3.003 (5)	173 (5)
C10—H10*C*···*Cg*2^iii^	0.98	2.83	3.723 (6)	152

Symmetry codes: (i) *x*+1/2, −*y*+1/2, *z*+1/2; (ii) *x*−1/2, −*y*+1/2, *z*−1/2; (iii) *x*, −*y*+1, *z*−1/2.
